# When Depression Breeds Rejection Rather Than Compassion: Disagreeableness, Stigma, and Lack of Empathic Concern Among Support Providers

**DOI:** 10.3389/fpsyt.2021.594229

**Published:** 2021-06-29

**Authors:** Myriam Mongrain, Ariel Shoikhedbrod

**Affiliations:** Department of Psychology, York University, Toronto, ON, Canada

**Keywords:** depression, agreeableness, stigma, empathy, compassion, rejection, interpersonal circumplex, social support

## Abstract

Past research has shown that the close relationships of depressed individuals are often characterised by rejection rather than compassion. The goal of this research was to broaden interpersonal models of depression by investigating the reports of support providers themselves. Individual differences, including disagreeableness, stigmatic beliefs about depression, and empathic concern were measured. These were examined in relation to reported interpersonal behaviours toward a significant other who was currently depressed. A cross-sectional design was used in an undergraduate (*N* = 312) and community sample (*N* = 296). Disagreeable individuals reported less compassionate and more rejecting behaviours toward depressed significant others based on an interpersonal circumplex model of social support. Serial mediation models further indicated that the associations between disagreeableness and rejecting behaviours reported by providers were mediated by stigma and lower empathic concern. The current studies shed light on how the personality, attitudes and emotions of support providers influence the level of compassion expressed toward depressed individuals.

## Introduction

Compassion involves a deep awareness of the suffering of another coupled with the wish to relieve it. The empirical literature supports the personal benefits accrued from compassionate responding. For example, providers of social support have been shown to live longer (1) and respond better to stress (2, 3). Compassion comes with other adaptive advantages (4) such as increases in subjective well-being (5), and greater meaning in life (6). Meta-analytic reviews of experimental interventions designed to increase compassionate responding have confirmed the causal relationship between kindness and subjective well-being (7, 8). Despite these redemptive qualities, compassion can be conspicuously absent in some interpersonal situations involving a significant other who is experiencing emotional suffering. For example, depressed individuals are more likely to report rejection rather than compassion from others in their immediate social environment (9, 10). The current research examined the characteristics of social environments marked by an absence of compassion toward a significant other who is depressed. We examined the personality, attitudes, and emotions reported by support providers as they relate to compassionate vs. rejecting behaviours toward depressed individuals.

Interpersonal models of depression have documented the ways in which depressed individuals can contribute to the negative interactional cycles in their close relationships (11, 12). For example, Coyne's interactional theory of depression (13, 14) proposed that depressed individuals' deep insecurities around their worth and lovability lead to repetitive and persistent attempts to get reassurance from loved ones. These attempts rarely bring lasting comfort, and significant others ultimately become frustrated and irritated with the depressed person (13, 14). Over time, this ultimately leads to rejection which in turn has been related to the hastened onset of a depressive episode as well as the exacerbation and perpetuation of symptoms (11, 15–17). This model has enjoyed considerable support in terms of the noxious effect of excessive reassurance seeking as a contributor to rejection in depressed populations (12, 15, 18). Decades of research were synthesised in a meta-analysis of 38 studies demonstrating a moderate association between depression, excessive reassurance seeking, and interpersonal rejection (19).

Other interpersonal models have emphasised social skills deficits and non-verbal behaviours associated with depressive states that are aversive to others and contribute to negative interpersonal outcomes [see (11)]. For example, depressed individuals speak in a slow, monotone voice, engage in less eye contact, and are less animated in their communication (20). They self-disclose negative information about themselves at inopportune times and may even solicit criticism from others [12, (15)]. The negative feedback seeking contributes to the maintenance of low self-esteem through self-verification mechanisms [see (21)]. These dynamics have received some support in the literature and describe how depressed individuals' behavioural patterns can contribute to their rejection in close relationships.

A caveat in this literature is the relative neglect of social support providers themselves and the ways in which their personality and beliefs may independently contribute to rejecting outcomes. Preliminary evidence suggests that characteristics of providers, such as intolerance and lack of empathy, play a significant role in the depression-rejection link. For example, Joiner et al. (18) reported that depressed males who were high on reassurance seeking and low in self-esteem were rejected by roommates who were intolerant and unempathetic, but not by roommates who were supportive. This suggests that characteristics of support providers may independently contribute to the erosion of compassion in the relationships of depressed individuals. The current study examines individual differences among support providers that could be associated with compassionate and rejecting behaviours toward a depressed significant other.

Trait agreeableness may be the single most important personality dimension influencing prosocial tendencies and the expression of compassionate behaviours (22–24). Agreeable individuals are described as tender, loving, and compassionate (25). They report greater empathic concern and are consequently more likely to help others in need (26, 27). This personality variable has been associated with a range of prosocial outcomes, including management of interpersonal conflict, communal concerns, as well as tolerance and cooperation in peer relationships [see (28, 29)]. Other research has shown that agreeableness is related to less prejudicial actions against out-group members [for a comprehensive review, see (30)].

Conversely, those reporting low levels of agreeableness (referred to as disagreeableness from here onward), have been described as antagonistic, indifferent to others, and hostile in daily life [see (28, 31, 32)]. Disagreeable individuals are prone to conflict and place personal interests ahead of others (23, 24). They have been shown to have a higher and stable frequency of quarrelsome behaviours across contexts and over time (33). Also problematic in marital relationships, disagreeableness entails poor conflict resolution abilities and dissatisfaction in those relationships (34, 35). Finally, disagreeableness has been related to stigmatisation and higher prejudicial biases against traditional target groups, as well as out-groups more generally [see (36, 37)].

Stigma entails blaming the victim for their emotional difficulties and not fully accepting them with those difficulties (38). Various forms of mental illness, including depression, can be seen as abhorrent and deviant conditions leading to prejudicial and discriminatory behaviours toward stigmatised victims (38). Stigmatised individuals can feel devalued and rejected as members of society (39). Personal stigma has been related to a lower likelihood of seeking help, indirect attempts at seeking support, and rejection in familial relationships (40). No study has yet examined the role of stigma as it relates to disagreeableness in the context of close relationships. The current study examines the relationship between disagreeableness, self-reported stigmatic attitudes, and supportive behaviours toward a significant other who was depressed.

A lack of empathy often accompanies the stigma directed toward individuals with mental illness (41). According to Batson's empathy-altruism hypothesis (2011), empathic concern is the immediate precursor to compassionate action. Empathic concern involves “other-oriented emotions congruent with the perceived welfare of someone in need” (p. 80). For empathic concern to produce altruistic motivation, one must value the other's welfare and see them as deserving (42, 43). As such, stigmatic attitudes impede on empathic processes by devaluing the deservingness of depressed individuals, amounting in rejection rather than compassion.

Compassionate and rejecting responses toward depressed targets can be captured with a circumplex model of supportive behaviours (44). The interpersonal circumplex (44–46) is a well-established nomological system which effectively captures the full range of interpersonal behaviours including compassion and rejection (47, 48). It is represented as a circular array of variables organised around two orthogonal axes labelled as dominance and love (44, 49). By creating a circular space that blends dominant (e.g., directive vs. avoidant) and loving (e.g., nurturant vs. critical) manifestations of support, the circumplex is ideal for the documentation of interpersonal behaviours expressed toward depressed targets. The blends of dominance and love provide a nuanced description of the types of responses that are compassionate and helpful (i.e. nurturant/loving and directive/dominant). Rejecting and hurtful responses are reflected by the opposite poles of the same axes (e.g., critical/hostile and avoidant/submissive).

Formal definitions of compassion can help locate its position along the axes of the interpersonal circumplex. As traditionally defined, compassion is comprised of three components: (1) Perceiving the other in need, (2) Feelings of empathic concern, or sympathy toward the other, and (3) Wanting to ease their suffering through concrete action (4, 50, 51). These components of compassion imply nurturing and directive actions aimed at reducing the others' suffering. In circumplex language, this involves a blend of more loving and dominant behaviours. Rejection, on the other hand, is located on the opposing quadrant of the circumplex reflecting critical and avoidant behaviour (i.e., low love and low dominance).

In a recent application of the circumplex model in close relationships, Lizdek et al. (52) showed that depressive symptoms in husbands and wives were associated with unique interpersonal dynamics during a conflict resolution task. More specifically, depression in wives was associated with dominant behaviours during the interaction, while husbands engaged in more submissive behaviours. Conversely, depression in husbands was associated with less loving behaviours over the course of the conflict resolution task for both partners. This study demonstrates how the love and dominance axes of the circumplex can provide nuanced insights into the interpersonal dynamics between depressed individuals and their significant others.

Other interpersonal constructs have been situated within the nomological net of the circumplex. Agreeableness is strongly related to interpersonal behaviours and maps onto the loving axis of the circumplex (53, 54). Depending on its measurement, agreeableness can vary in levels of dominance, with facets such as modesty falling in the submissive quadrant (53). Conversely, disagreeableness may be found in the hostile/dominant quadrant of the circumplex. Similarly, empathic concern has been associated with loving and some degree of dominance in the supportive actions captured on the interpersonal circumplex (44). Love and dominance have not been explored within the context of supportive behaviours provided to depressed individuals specifically. The interpersonal circumplex was adopted in the current studies as a framework for capturing the roles of disagreeableness, stigma and empathic concern in relation to compassionate and rejecting behaviours reported toward depressed individuals.

### Current Research

Decades of research on the interactional theory of depression suggest that depressed individuals burden their significant others with excessive reassurance seeking and or negative feedback seeking. There is evidence that these behaviours effectively erode support from the immediate social environment and ultimately lead to rejection (12). Provider characteristics contributing to the negative interpersonal outcomes in the relationships of depressives have been neglected in the empirical literature. The current work examines disagreeableness in providers and their reported levels of stigma and empathic concern toward a depressed significant other. These variables were expected to predict compassionate and rejecting forms of support as reported by providers.

Compassionate and rejecting forms of support were examined along the loving and dominant axes of the circumplex. Compassion was operationalized as high scores on love (e.g., nurturant) and dominance (e.g., directive). Rejection was operationalized as lower scores on the love (e.g., critical) and dominance (e.g., avoidant) axes. The main predictions were that stigmatising attitudes about depression and low empathic concern would mediate the relationship between disagreeableness and supportive behaviours reported toward a depressed target. The specific hypotheses are as follows:

H1: Disagreeableness in providers will be associated with greater stigma, lower empathic concern, as well as less compassionate and more rejecting forms of support on the circumplex.
H2: Stigma will mediate the relationship between disagreeableness and rejecting behaviours.
H3: Empathic concern will mediate the relationship between disagreeableness and rejecting behaviours.
H4: Stigma and empathic concern will serially mediate the relationship between disagreeableness and rejecting behaviours.

Two studies were conducted to test these predictions using an undergraduate (Study 1) and community sample (Study 2).

## Materials and Methods

### Study 1

#### Method

##### Participants

Undergraduate students (*N* = 349) were recruited from a Canadian university through an undergraduate participant pool. We advertised for individuals with a significant other (i.e., friend, family member, romantic partner) who was currently depressed. Based on Schönbrodt and Perugini (55) recommendation of sample sizes approximating *N* = 250 for stable correlation estimates, we oversampled to account for anticipated data exclusions. We omitted participants whose significant other was not depressed (*n* = 5), those who failed attention checks on the Conscientious Responders Scale (CRS; 50; *n* = 11), or had open-ended responses suggesting ineligible or disingenuous responding (*n* = 2). Incomplete responders on any of the measures were also omitted (*n* = 13) along with those who asked to not have their data included in the study (*n* = 6).

The final sample for Study 1 included 312 participants. They were mostly women (71.5% female) with an average age of 20.13 years (*SD* = 4.29). Participants identified themselves as White (27.24%), South Asian (23.72%), Middle Eastern (16.35%), Black (12.50%), South East Asian (5.77%), Mixed (4.49%), Latin American (3.53%), and South American (0.96%). They also reported being mildly depressed at the time of the study with an average score of 11.72 (*SD* = 6.07) on the short-form Centre for Epidemiological Studies Depression Scale [CESD-10; (56)].

Participants also completed demographic questions regarding their significant other who was currently depressed. These significant others were on average 24.85 years old (*SD* = 10.96) and 61.28% were female. They consisted of friends (60.90%), family (31.41%), romantic partners (5.13%), and others (2.56%). Participants had known the depressed target for over 9 years (*M* = 9.42, SD = 7.13), and interacted with them daily (40.71%), a few times a week (33.65%), once a week (8.33%), every other week (6.41%), once a month (6.09%), less than once a month (3.85%), and not at all (0.96%)^1^.

The depressed status of the significant other was further confirmed with two measures. One question, “How depressed is this person?,” received an average rating of 4.57 (*SD* = 1.13) out of a 7- point scale (1 = *not at all*, 7 = *very much so*). Additionally, a scale was created from the criteria listed in the Diagnostic and Statistical Manual of Mental Disorders [DSM-5; (57)] under Major Depressive Disorder^2^. Significant others were described as having an average of 5.11 (*SD* = 1.95) out of 8 symptoms for major depression, suggesting they were perceived by participants as clinically depressed.

##### Procedure

Participants were then provided a link to Qualtrics, an online survey platform, to complete the questionnaires in the study. Informed consent was obtained followed by demographic questionnaires about themselves, the depressed target, and general characteristics of their relationship with the target (i.e., type, length, and frequency). Participants then responded to questionnaires about their empathic concern and interpersonal behaviours toward the depressed target followed by personality measures of disagreeableness and depression stigma. Participants were debriefed at the end of the study and credited for their participation.

##### Measures

###### Ten Item Personality Inventory

[TIPI; (58)]. The TIPI is a brief and widely used measure of the five-factor model of personality (i.e., Openness, Conscientiousness, Extraversion, Agreeableness, and Neuroticism). The agreeableness subscale consists of 2 items (e.g., I see myself as critical, quarrelsome; I see myself sympathetic, warm) rated on a 7-point scale from 1 (*disagree strongly*) to 7 (*agree strongly*). The items were reverse scored to reflect disagreeableness among support providers. Although the TIPI has weaker psychometric properties relative to lengthier five factor personality measures, it demonstrates favourable factor structure and convergent validity (59). The two items were moderately correlated (*r* = 0.31) in the current sample.

###### Centre for Epidemiological Studies Depression Scale, Short Form

[CES-D 10; (56)]. The CES-D 10 was derived from the original CESD (60) and asked participants to rate the frequency of their depressive symptoms during the past week from 1 = *rarely or none of the time* to 4 = *most or all of the time*. The cut-off for clinically significant levels of depression is 10 (56). Its psychometric properties are robust relative to the widely used 20-item CES-D scale (61, 62). The CESD demonstrated acceptable internal consistency in the current sample (α = 83).

###### Depression Stigma Scale

[DSS; (39)]. The DSS was designed to assess stigmatising attitudes toward depression. A subscale of the DSS was used to measure participants' self-endorsed level of stigma. The scale is comprised of 9 items (e.g., “People with depression could snap out of it if they wanted”) assessed on a 7-point scale, ranging from 1 (*strongly disagree*) to 7 (*strongly agree*). The DSS has shown moderate to high internal consistency (39, 63), and moderate test–retest reliability (39). The DSS demonstrated acceptable internal consistency in the current sample (α = 83).

###### Empathic Concern Scale

[ECS; (64)]. The ECS captures the affective qualities of empathy that precede compassionate action. Participants reported the extent to which they felt empathic toward their depressed significant other. Six adjectives (e.g., sympathetic, soft-hearted, warm, compassionate, tender, and moved) were rated from 1 (*Not at all*) to 7 (*Extremely*). The ECS had acceptable internal consistency in the current sample (α = 89), which is consistent with past research (65).

###### Support Actions Scale Circumplex

[SAS-C; (44)]. The SAS-C is comprised of 64 items measuring supportive behaviours along the love and dominance axes of the circumplex. Participants reported the extent to which they engaged in loving and dominant behaviours toward the depressed target. The items can be further divided into 8 octants representing combinations of love and dominance as described by interpersonal circumplex models (e.g., 42). Going counter-clockwise on the circumplex starting at the top of the dominance axis, the octants included: PA/Directive (α = 0.76, e.g., “I would tell them to let me help with their problem”), BC/Arrogant (α = 0.77, e.g., “I would advise them to pay attention to what I have to say”), DE/Critical (α = 0.78, e.g., “Remind them that people sometimes get what they deserve”), FG/Distancing (α = 0.79, e.g., “Tell them I don't want to get involved”), HI/Avoidant (α = 0.70, e.g., “I would shy away from making suggestions”), JK/Deferential (α = 0.62, e.g., “I would not give my opinions unless asked”), LM/Nurturant (α = 0.76, e.g., “I would give them a hug”), and NO/Engaging (α = 0.83, e.g., “I would try to involve them in social activities”). Items are rated on a 7-point scale ranging from 1 (*I definitely wouldn't do this*) to 7 (*I definitely would do this*). The psychometric properties of the scale have been well-established (44).

#### Results Study 1

In the preliminary analyses, we examined the descriptive properties of the interpersonal circumplex as well as the correlations between disagreeableness, depression stigma, empathic concern, and supportive behaviours on the SAS-C (see H1). After ruling out potential covariates, we ran the mediation analyses to test our main hypotheses (H2-H4).

##### Preliminary Analyses

**Figure 1:**
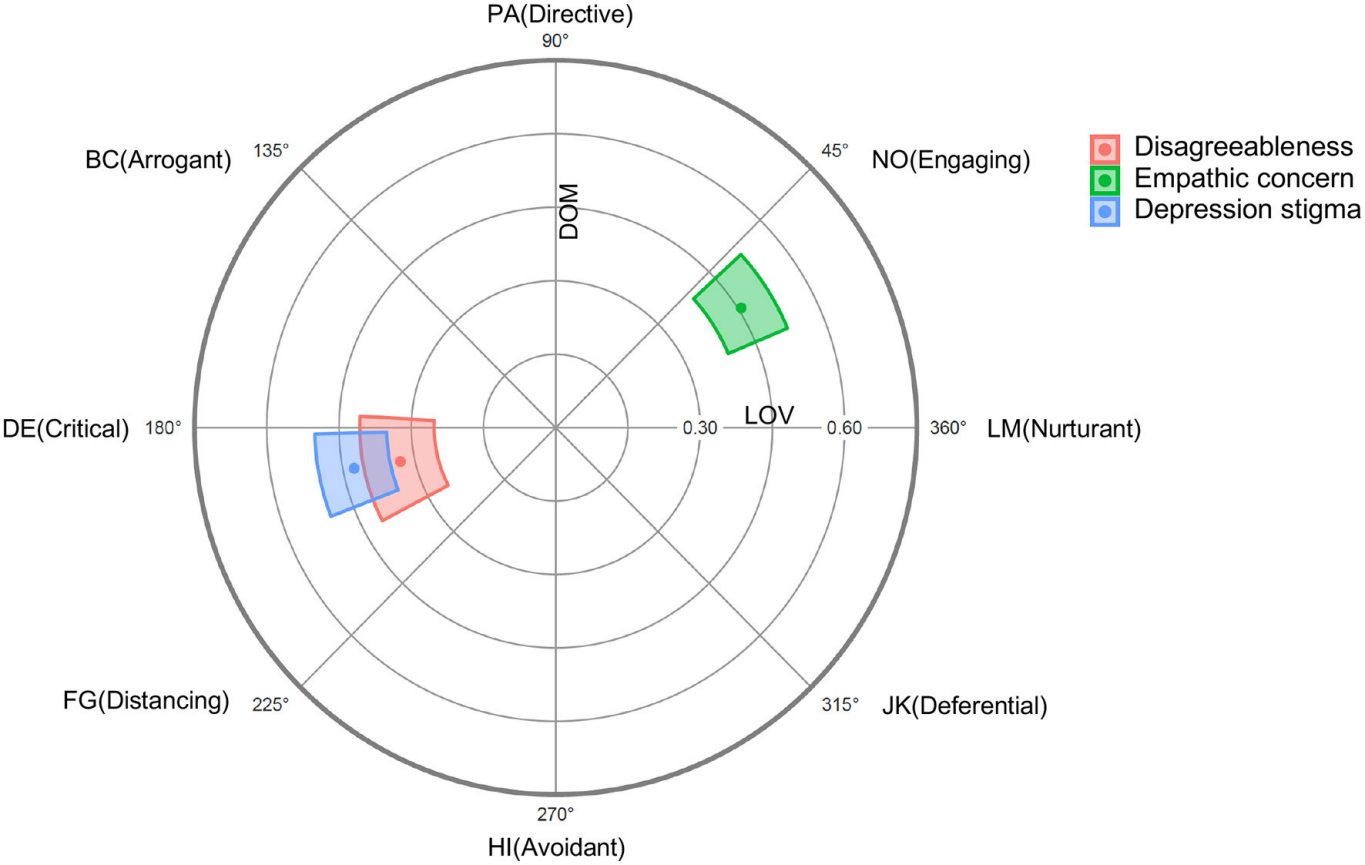
Radar chart of Disagreeableness, Empathic concern and Depression stigma projected on to the SAS-C in Study 1. Dots represent mean values and coloured regions represent bootstrapped 95% confidence intervals [computed and plotted using the circumplex package for R; (67)].

The Structural Summary Method [SSM, (47, 66)] was employed to examine how well-disagreeableness, depression stigma, and empathic concern adhered to a circumplex configuration. Figure 1 provides a visual representation of the SSM parameters, and Table 1 includes the SSM statistics. The results indicate an excellent fit to a circumplex configuration for all the criterion variables. This suggests that the profiles for disagreeableness, stigma and empathic concern were prototypical in nature, allowing for the interpretation of the other SSM parameters. Amplitude values (see Table 1) showed that disagreeableness, stigma, and empathic concern were uniquely related to specific social support behaviours. The angular displacement value for disagreeableness ranged from 177 to 209 degrees (see Table 1) associating high scores on disagreeableness with critical (DE) and distancing (FG) behaviours toward depressed targets. Stigma was similarly located in the hostile and submissive quadrant of circumplex with angular displacement values ranging from 180 to 200 degrees. The angular displacement values for empathic concern, on the other hand, ranged from 24 to 43, positioning this variable between the nurturant (LM) and engaging (NO) octants of the SAS-C (see Figure 1). Elevation parameters were low, indicating that the criterion variables were not associated with social support actions generally [see (68) for parameter interpretation]. Overall, these results provide confidence that the profiles for disagreeableness, depression stigma, and empathic concern are appropriate for a circumplex interpretation.

**Table 1:** Structural summary parameters with 95% confidence intervals in Study 1.

**Profile**	**Love**	**Dominance**	**Elevation**	**Amplitude**	**Displacement**	**Fit**
Disagreeableness	−0.32 [−0.40, −0.23]	−0.08 [−0.16, 0.02]	−0.03 [−0.08, 0.02]	0.32 [0.24, 0.41]	193.4 [177.0, 208.5]	0.98
Depression stigma	−0.43 [−0.50, −0.35]	−0.08 [−0.16, −0.00]	0.05 [0.00, 0.10]	0.43 [0.36, 0.51]	190.5 [180.2, 200.3]	0.98
Empathic concern	0.38 [0.31, 0.45]	0.26 [0.17, 0.34]	0.11 [0.06, 0.15]	0.46 [0.39, 0.53]	33.8 [23.7, 43.4]	0.99

The descriptive statistics and zero-order correlations between the study variables are presented in Table 2. Disagreeableness was negatively related to loving support on the SAS-C (*r* = −0.41, *p* < 0.001) but not with dominant support in Study 1 (*r* = 0.10, *p* = 0.084). Similarly, depression stigma was associated with less loving support (*r* = −0.56, *p* < 0.001). Empathic concern, however, was positively associated with both love (*r* = 0.50, *p* < 0.001) and dominance (*r* = 0.35, *p* < 0.001) indicating active engagement for participants reporting greater empathy. Disagreeableness was correlated with both depression stigma (*r* = 0.37, *p* < 0.001) and lower empathic concern (*r* = −0.29, *p* < 0.001). Finally, stigma correlated negatively with empathic concern (*r* = −0.34, *p* < 0.001).

**Table 2:** Descriptive statistics and correlations in Study 1.

	**1**	**2**	**3**	**4**	**5**	**6**	**7**	**8**	**9**	**10**	**11**	**12**	**13**	**14**	**15**	**16**
1. Age	—															
2. Gender	0.05	—														
3. CESD−10^a^	−0.10	0.21^**^	—													
4. Disagreeableness^b^	−0.05	−0.21^**^	0.03	—												
5. Depression stigma^c^	0.02	−0.26^**^	−0.08	0.37^**^	—											
6. Empathic concern^d^	0.07	0.19^**^	0.00	−0.29^**^	−0.34^**^	—										
7. Love^e^	0.01	0.30^**^	0.09	−0.41^**^	−0.56^**^	0.50^**^	—									
8. Dominance^e^	0.00	−0.00	−0.12^*^	−0.10	−0.10	0.35^**^	0.15^*^	—								
9. Directive (PA)^f^	0.03	−0.00	−0.07	−0.04	−0.02	0.33^**^	0.10	0.77^**^	—							
10. Arrogant (BC)^f^	0.09	−0.12^*^	−0.04	0.17^**^	0.29^**^	0.07	−0.42^**^	0.51^**^	0.65^**^	—						
11. Critical (DE)^f^	0.03	−0.30^**^	−0.12^*^	0.32^**^	0.53^**^	−0.32^**^	−0.78^**^	−0.10	0.11	0.45^**^	—					
12. Distancing (FG)^f^	0.08	−0.18^**^	0.10	0.22^**^	0.39^**^	−0.35^**^	−0.59^**^	−0.53^**^	−0.17^**^	0.22^**^	0.57^**^	—				
13. Avoidant (HI)^f^	0.05	0.03	0.08	0.06	0.10	−0.09	−0.06	−0.71^**^	−0.20^**^	−0.02	0.18^**^	0.44^**^	—			
14. Deferential (JK)^f^	0.11^*^	0.15^**^	0.11	−0.15^*^	−0.12^*^	0.19^**^	0.45^**^	−0.42^**^	0.01	−0.09	−0.09	0.17^**^	0.53^**^	—		
15. Nurturant (LM)^f^	0.06	0.21^**^	0.10	−0.35^**^	−0.35^**^	0.50^**^	0.80^**^	0.21^**^	0.29^**^	−0.06	−0.41^**^	−0.26^**^	0.03	0.46^**^	—	
16. Engaging (NO)^f^	0.10	0.17^**^	−0.01	−0.32^**^	−0.34^**^	0.57^**^	0.66^**^	0.58^**^	0.62^**^	0.21^**^	−0.28^**^	−0.36^**^	−0.17^**^	0.29^**^	0.73^**^	—
M	20.13	—	11.73	4.97	2.33	5.25	−0.00	0.01	4.51	3.17	2.09	2.87	3.27	4.51	5.69	5.46
SD	4.29	—	6.07	1.07	0.82	1.17	0.93	0.87	1.06	1.07	1.00	1.01	0.90	0.90	0.90	1.01

*Sample size for each correlation ranged between 297 and 312 participants due to missing data*.

a *Centre for Epidemiological Studies Depression Scale, Short Form (56). Reverse scored agreeableness factor score from the Ten Item Personality Inventory (58)*.

c *Empathic Concern Scale (64)*.

d *Depression Stigma Scale (39)*.

e *Standardised scores for the Love and Dominance axes of the Support Actions Scale Circumplex (44)*.

f *Octant scores from the Support Actions Scale Circumplex (44)*.

* *p < 0.05*,

** *p < 0.01*.

Female providers reported lower disagreeableness (*r* = −0.21, *p* < 0.001), lower levels of depression stigma (*r* = −0.26, *p* < 0.001), and greater empathic concern (*r* = 0.19, *p* = 0.001). Women also reported providing more loving support (*r* = 0.30, *p* < 0.001). Additionally, depression in providers was negatively associated with dominant support (*r* = −0.12, *p* = 0.040). Controlling for gender and depression in participants did not significantly alter the results (see Supplementary Materials).

##### Serial Mediation Analyses

It was hypothesised that the effect of disagreeableness on the type of support provided would be explained by depression stigma and a lack of empathic concern toward depressed targets. Given the non-significant relationship between disagreeableness and dominance in Study 1, the first condition for mediation was not met and was not explored further (69). There was, however, a strong and negative relationship between disagreeableness and loving support reported toward targets. Consequently, the serial mediation analyses were justified for social support outcomes on the love axis.

**Figure 2:**
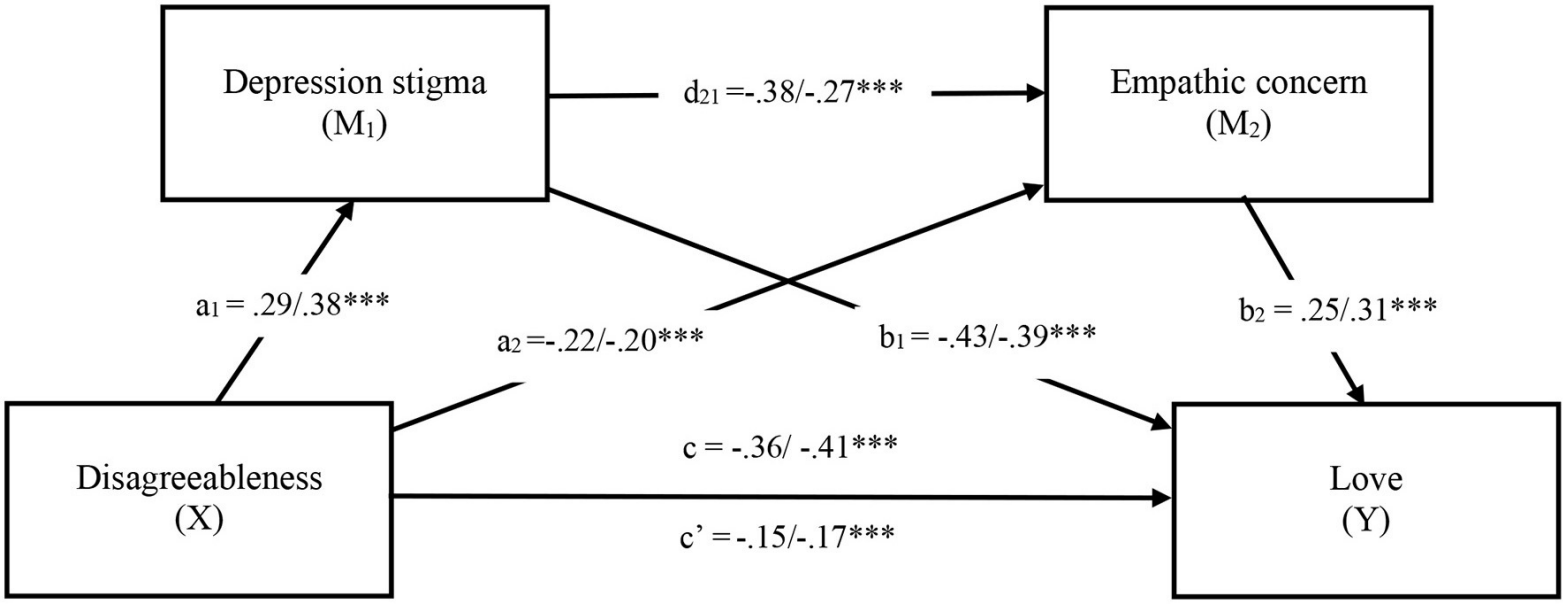
Serial mediation model in Study 1. Note. Values shown reflect unstandardized/standardised coefficients. *p < 0.05, **p < 0.01, ***p < 0.001. c = Total effect, c′ = Direct effect.

The analyses were conducted using Hayes' (70) SPSS macro PROCESS (Model 6) with 95 % bias corrected confidence interval (CI) based on 5,000 bootstrap samples. Figure 2 depicts the unstandardized and standardised path coefficients in a serial mediation model where disagreeableness (X) is modelled as effecting love (Y) through depression stigma (M_1_) and empathic concern (M_2_). The total effect of disagreeableness on love, without the mediators in the model, was significant (*b* = −0.36, *SE* = 0.05, *t* = −7.74, *p* < *0.0*01, 95% CI [−0.45, −0.27]). When depression stigma and empathic concern were entered as mediators, the direct effect of disagreeableness on love was attenuated but remained significant (*b* = −0.15, *SE* = 0.04, *t* = −3.55, *p* < *0.0*01, 95% CI [−0.23, −0.07]). The total indirect effect of the model was also significant suggesting partial mediation (see Table 3).

**Table 3:** Total, direct, and indirect effects of disagreeableness (X) on love (Y) through depression stigma (M1) and empathic concern (M2) in Study 1.

	**Effect**	***SE***	***t***	***p***	**LL**	**UL**
Total effect	−0.36	0.05	−7.74	<0.001	−0.45	−0.27
Direct effect	−0.15	0.04	−3.55	<0.001	−0.23	−0.07
Total indirect effect	−0.21	0.03			−0.27	−0.15
Indirect effect (X→*M*_1_→Y)^a^	−0.13	0.03			−0.18	−0.08
Indirect effect (X→*M*_2_→Y)^a^	−0.05	0.02			−0.09	−0.02
Indirect effect (X→*M*_1_→*M*_2_→Y)^a^	−0.03	0.01			−0.05	−0.01

a *based on 5,000 bootstrap samples. LLCI, Lower Limit 95% Confidence Interval; ULCI, Upper Limit 95% Confidence Interval*.

The specific indirect effects of stigma and empathic concern, individually and jointly, were examined next. As shown in Figure 2, Stigma and empathic concern, on their own, significantly mediated the association between disagreeableness and lower levels of loving support. Further, the serial effect suggest that stigma followed by reduced empathic concern both explain the relationship between disagreeableness less loving support (see Figure 2).

Study 1 provided preliminary evidence for the hypothesised effects (H1-H4) of provider characteristics on supportive behaviours. Disagreeable providers reported being more critical and distant toward depressed targets. The effect of disagreeableness was particularly salient along the love axis of the circumplex. Disagreeableness was correlated with stigmatic attitudes and less empathic concern toward depressed targets. Depression stigma and empathic concern individually and serially mediated the relation between disagreeableness and less loving support. In Study 2, we sought to systematically replicate these findings in a community sample.

### Study 2

#### Method

##### Participants

We recruited community members from “Prolific Academic,” which is a UK based crowdsourcing platform. There were 350 participants initially screened confirming their relationship with a significant other who was currently depressed. Of those, 303 enrolled in the study. Seven participants were excluded for the following reasons: responding that their significant other was not depressed (*n* = 1), selecting to not include their data in the study (*n* = 1), and open-ended responses suggesting ineligible or disingenuous responding (*n* = 5). The remainder of the participants passed the Conscientious Responders Scale [CRS; (71)].

The final sample consisted of 296 adults (62% female) with an average age of 31.81 years (*SD* = 11.39). Participants were predominantly from English-speaking countries: United Kingdom (59.12%), United States (18.24%), Canada (14.53%), and others (8.11%). Participants' ethnic backgrounds were primarily White (78.04%), followed by Black (5.41%), Mixed (4.39%), South Asian (4.39%), East Asian (3.72%), South East Asian (2.36%), Latin American (1.01%), and Middle Eastern (0.68%). Participants' employment status ranged from being employed full-time (46.60%), employed part-time (19.93%), students (11.82%), unemployed (10.47%), students with part time job (6.08%), retired (2.36%), and at home caregivers (2.36%). Education level ranged from an in-progress college education (19.93%), high school (18.58%), master's degree (13.51%), college diploma or associate degree (11.15%), doctoral degree (2.70%), and less than high school (0.34 %). Participants were single (34.46%), married (30.74%), in a committed relationship (3.72%), common law (3.72%), divorced (2.70%), in a casual relationship (2.70%), separated (1.01%), or widowed (0.34%). The mean score on the CESD-10 (56) was 11.03 (SD = 6.78), indicating that participants reported mild levels of depressive symptoms at the time of the study.

Participants completed a demographics questionnaire describing their depressed significant other. These depressed targets were on average 34.67 years old (*SD* = 14.97) and slightly more than half (57.78%) were female. They consisted of friends (46.96%), family members (30.41%), romantic partners (18.31%), among others (4.05%). Participants had known the depressed person for almost 11 years (*M* = 10.85 years; SD = 14.97) and interacted with them daily (43.9%), a few times a week (33.45%), once a week (8.45%), every other week (6.76%), once a month (3.72%), less than once a month (3.38%), or not at all (0.34%)^3^.

The depressed status of the significant other was confirmed with two measures. One item, “How depressed is this person?,” received an average rating of 4.79 out of 7 (SD = 0.92) (1 = *not at all*, 7 = *very much so*), indicating that significant others were perceived as being moderately depressed. Significant others were also described as having 5.20 (SD = 1.76) out of the 8 symptoms based on the scale of DSM-5 criteria for major depressive disorder.

##### Procedure

Participants were initially screened on Prolific for the presence of a significant other who was currently depressed using the same descriptive summary as in Study 1. Those who confirmed their eligibility were then provided a link to a Qualtrics survey, where they engaged in the same procedure as in Study 1. Participants were debriefed at the end of the study and compensated £2.27 for their participation.

##### Measures

###### Big Five Inventory

[BFI; (72)]. The BFI is a 44-item questionnaire measuring the five-factor model of personality (i.e., openness, conscientiousness, extraversion, agreeableness, and neuroticism). The BFI provides a psychometrically stronger alternative to the TIPI (58). The agreeableness subscale of the BFI consists of 9 items (e.g., “Tends to find faults with others”) rated on a 5-point scale (1 = *Disagree Strongly*, 5 = Agree Strongly). The items were reverse scored to reflect disagreeableness among support providers. The disagreeableness subscale of the BFI had acceptable internal consistency in the current sample (α = 0.78).

###### Centre for Epidemiological Studies Depression Scale, Short Form

[CES-D 10; (56)]. Similar to Study 1, CES-D 10 was used to measure depression among support providers. The CESD demonstrated acceptable internal consistency in the current sample (α = 0.94).

###### Depression Stigma Scale

[DSS; (39)]. Similar to Study 1, the DSS was used to measure the extent to which support providers endorsed stigmatic beliefs about depression. The DSS demonstrated acceptable internal consistency in the current sample (α = 0.80).

###### Empathic Concern Scale

[ECS; (64)]. Similar to Study 1, the ECS was used to measure the extent to which support providers felt empathic toward their depressed significant other. The ECS had acceptable internal consistency in the current sample (α = 93).

###### Support Actions Scale Circumplex

[SAS-C; (44)]. Similar to Study 1, the SAS-C was used to measure the loving and dominant forms of support toward depressed significant others as reported by support providers. The octants of the SAS-C (PA α = 0.79, BC α = 0.79, DE α = 0.80, FG α = 0.80, HI α = 0.82, JK α = 0.70, LM α = 0.77, NO α = 0.83) all demonstrated acceptable internal consistency in the current sample.

#### Results Study 2

In the preliminary analyses, we examined the descriptive properties of the interpersonal circumplex as well as the correlations between disagreeableness, depression stigma, empathic concern, and supportive behaviours on the SAS-C (see H1). After ruling out potential covariates, we ran mediation analyses to test our main hypotheses (H2-H4).

##### Preliminary Analyses

**Figure 3:**
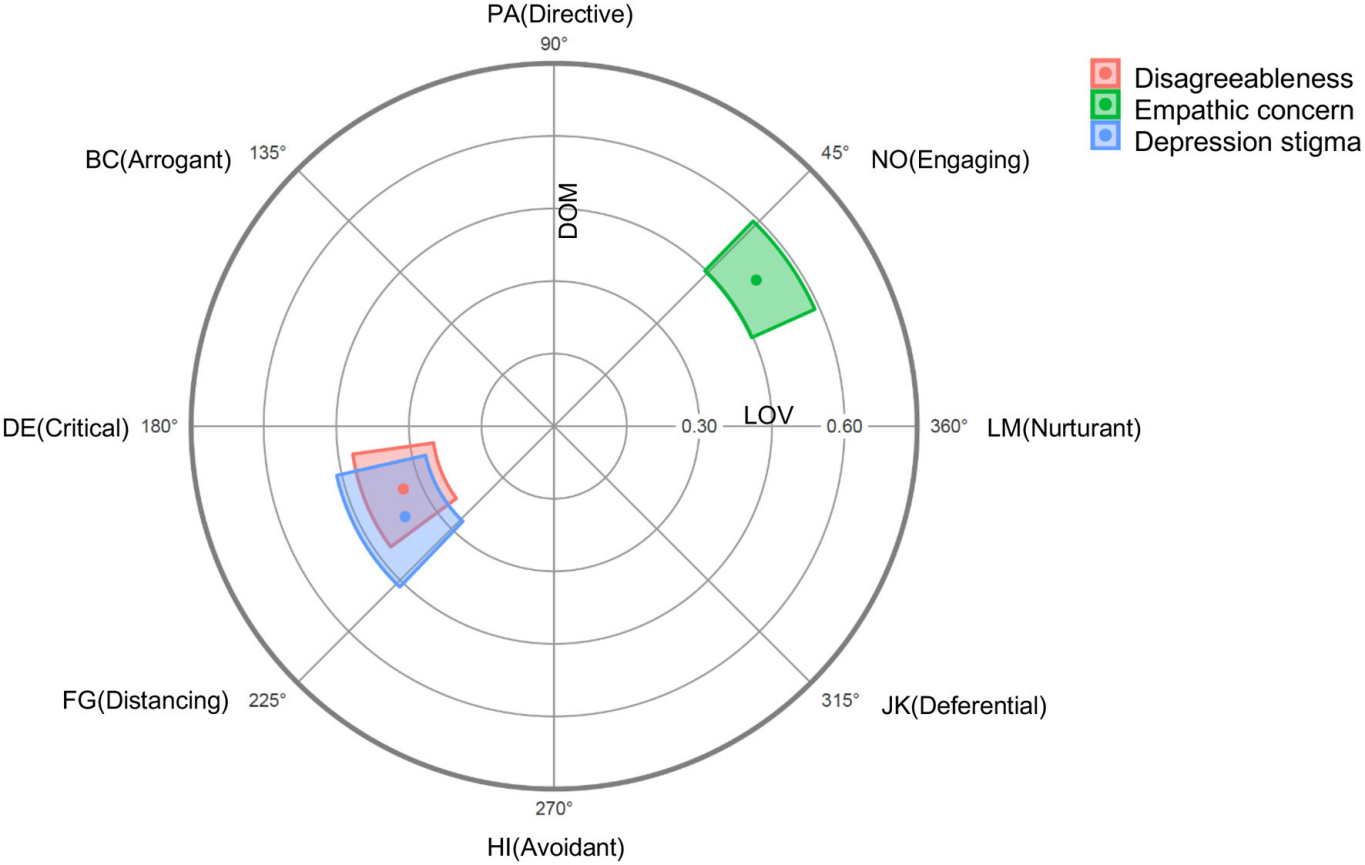
Radar chart of Disagreeableness, Empathic concern and Depression stigma projected on to the SAS-C in Study 2. Dots represent mean values and coloured regions represent bootstrapped 95% confidence intervals (computed and plotted using the circumplex package for R; (67).

The Structural Summary Method [SSM; (47, 66, 68)] was employed to examine the fit of our criterion variables to a circumplex configuration. SSM parameters were obtained to determine whether disagreeableness, stigma and empathic concern are well-represented within the two-dimensional space marked by love and dominance. The SSM statistics for Study 2 are presented in Table 4. The results indicate an excellent fit to a circumplex configuration for all the criterion variables. The profiles for disagreeableness, stigma and empathic concern were prototypical in nature, allowing for the interpretation of the other SSM statistics. Amplitude values (see Table 4) suggested that disagreeableness, stigma, and empathic concern were uniquely related to specific social support behaviours. As illustrated in Figure 3, the angular displacement value for disagreeableness ranged from 188 to 217 degrees, indicating that this personality variable was accompanied by a preponderance of critical (DE) and distancing (FG) behaviours toward depressed targets. Stigma was similarly located in the hostile and submissive quadrant of the circumplex, with values ranging from 191 to 226 degrees. The angular displacement values for empathic concern ranged from 24 to 43 degrees, positioning this variable between the nurturant (LM) and engaging (NO) octants of the SAS-C (see Figure 3). The elevation parameters further suggest that empathic concern was generally associated with more supportive behaviours (see Table 4). Overall, these SSM statistics provide confidence that the profiles for disagreeableness, depression stigma, and empathic concern are appropriate for a circumplex interpretation.

**Table 4:** Structural summary parameters with 95% confidence intervals in Study 2.

**Profile**	**Love**	**Dominance**	**Elevation**	**Amplitude**	**Displacement**	**Fit**
Disagreeableness	−0.32 [−0.40, −0.23]	−0.13 [−0.22, −0.04]	−0.05 [−0.10, 0.00]	0.34 [0.26, 0.43]	202.3 [188.1, 217.0]	0.97
Depression stigma	−0.30 [−0.38, −0.21]	−0.18 [−0.29, −0.06]	0.04 [−0.02, 0.09]	0.35 [0.25, 0.45]	210.8 [190.9, 225.7]	0.96
Empathic concern	0.41 [0.35, 0.47]	0.31 [0.20, 0.41]	0.18 [0.14, 0.23]	0.51 [0.44, 0.59]	36.5 [24.3, 46.5]	0.97

The descriptive statistics and zero-order correlations between the study variables are presented in Table 5. As predicted (H1), disagreeableness was negatively correlated with the love axis of the SAS-C (*r* = −0.42, *p* < 0.001). The association between disagreeableness and the dominance axis was small but significant (*r* = 0.17, *p* = 0.005). As predicted, depression stigma also predicted less loving (*r* = −0.40, *p* < 0.001), and less dominant (*r* = −0.23, *p* < 0.001) support. Empathic concern, on the other hand, was positively correlated with love (*r* = 0.54, *p* < 0.001) and dominance (*r* = 0.39, *p* < 0.001). Disagreeableness was associated with more stigma (*r* =0.22, *p* < 0.001) and less empathic concern (*r* = −0.30, *p* < 0.001). As expected, stigma was associated with less empathic concern (*r* = 0.34, *p* < 0.001).

**Table 5:** Descriptive statistics and correlations in Study 2.

	**1**	**2**	**3**	**4**	**5**	**6**	**7**	**8**	**9**	**10**	**11**	**12**	**13**	**14**	**15**	**16**
1. Age	—															
2. Gender	0.02	—														
3. CESD−10^a^	−0.25^**^	0.05	—													
4. Disagreeableness^b^	−0.23^**^	−0.08	0.37^**^	—												
5. Depression stigma^c^	0.10	−0.24^**^	−0.08	0.22^**^	—											
6. Empathic concern^d^	0.07	0.03	−0.04	−0.30^**^	−0.34^**^	—										
7. Love^e^	0.12^*^	0.19^**^	−0.02	−0.42^**^	−0.40^**^	0.54^**^	—									
8. Dominance^e^	−0.06	−0.02	−0.07	−0.17^**^	−0.23^**^	0.39^**^	0.16^**^	—								
9. Directive (PA)^f^	0.01	−0.00	−0.11	−0.19^**^	−0.13^*^	0.45^**^	0.17^**^	0.77^**^	—							
10. Arrogant (BC)^f^	−0.02	−0.14^*^	−0.02	−0.00	0.03	0.21^**^	−0.27^**^	0.63^**^	0.68^**^	—						
11. Critical (DE)^f^	−0.08	−0.26^**^	0.09	0.34^**^	0.36^**^	−0.24^**^	−0.77^**^	−0.10	−0.00	0.32^**^	—					
12. Distancing (FG)^f^	−0.04	−0.14^*^	0.06	0.31^**^	0.45^**^	−0.44^**^	−0.63^**^	−0.63^**^	−0.36^**^	−0.06	0.52^**^	—				
13. Avoidant (HI)^f^	0.13^*^	0.01	0.02	0.03	0.19^**^	−0.08	−0.00	−0.72^**^	−0.24^**^	−0.16^**^	0.14^*^	0.45^**^	—			
14. Deferential (JK)^f^	0.20^**^	0.06	0.04	−0.20^**^	−0.07	0.30^**^	0.54^**^	−0.42^**^	−0.02	−0.16^**^	−0.22^**^	0.07	0.56^**^	—		
15. Nurturant (LM)^f^	0.07	0.10	−0.00	−0.35^**^	−0.28^**^	0.61^**^	0.82^**^	0.20^**^	0.32^**^	0.00	−0.41^**^	−0.40^**^	0.07	0.52^**^	—	
16. Engaging (NO)^f^	0.03	0.02	0.00	−0.31^**^	−0.26^**^	0.61^**^	0.63^**^	0.67^**^	0.63^**^	0.33^**^	−0.28^**^	−0.56^**^	−0.26^**^	0.19^**^	0.65^**^	—
M	31.81	—	11.03	3.79	2.10	5.38	0.01	−0.00	4.49	3.11	1.78	2.65	3.31	4.73	5.81	5.53
SD	11.39	—	6.78	0.65	0.81	1.22	0.91	0.95	1.10	1.07	0.83	0.98	1.04	0.93	0.84	0.97

*Sample size for each correlation ranged between 288 and 295 participants due to missing data*.

a *Centre for Epidemiological Studies Depression Scale, Short Form (56)*.

b *Reverse scored agreeableness factor score from the Big Five Inventory (72)*.

c *Empathic Concern Scale (64)*.

d *Depression Stigma Scale (39)*.

e *Standardised scores for the Love and Dominance axes of the Support Actions Scale Circumplex (44)*.

f *Octant scores from the Support Actions Scale Circumplex (44)*.

* *p < 0.05*,

** *p < 0.01*.

Female providers endorsed lower stigma (*r* = −0.24, *p* < 0.001) and were more likely to provide loving support (*r* = 0.19, *p* = 0.001). Additionally, older people reported being less disagreeableness (*r* = −0.23, *p* < 0.001) and more loving in their support (*r* = 0.12, *p* = 0.049). Controlling for age and gender in the mediational analyses did not significantly alter the results (see Supplementary Materials).

##### Serial Mediation Analyses

**Figure 4:**
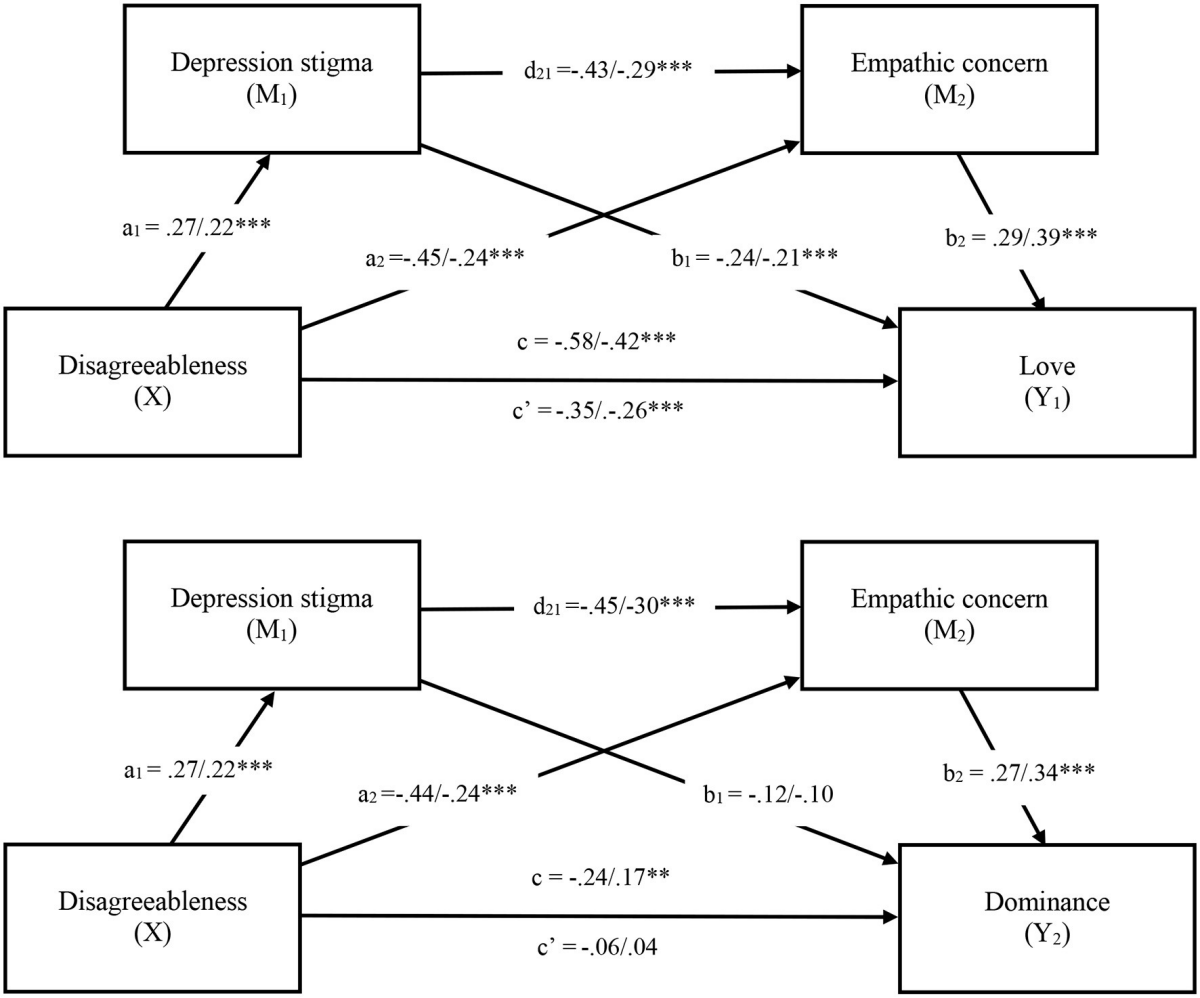
Serial mediation models in Study 2. Note. Values shown reflect unstandardized/standardised coefficients. *p < 0.05, **p < 0.01, ***p < 0.001. c = Total effect, c′ = Direct effect.

It was hypothesised that depression stigma and a lack of empathic concern would mediate the effect of disagreeableness on the love and dominance axes of the SAS-C. Figure 4 depicts a series of serial mediation models in which disagreeableness is modelled as effecting love and dominance through stigma and empathic concern. In the first model, the total effect of disagreeableness on love without the mediators in the model was significant (*b* = −0.58, SE = 0.07, *t* = −7.84, *p* < 0.001 95% CI [−0.73, −0.44]). When depression stigma and empathic concern were entered as mediators, the direct effect of disagreeableness on love was attenuated but remained significant (*b* = −0.35, SE = 0.07, *t* = −5.27, *p* < 0.001, 95% CI [−0.49, −0.22]). The total indirect effect of the model was also significant suggesting partial mediation (see Table 6). Both stigma and empathic concern individually mediated the association between disagreeableness and love in support of our hypotheses (H2-H3). The mediators also jointly accounted for the relationship between predictor and outcome (H4). That is, greater stigma and then lower empathic concern serially mediated the relationship between disagreeableness and less loving support (see Figure 4).

**Table 6:** Total, direct and indirect effects of disagreeableness (X) on love (Y_1_) and dominance (Y_2_) through depression stigma (M_1_) and empathic concern (M_2_) in Study 2.

	**Love**						**Dominance**					
	**Effect**	***SE***	***t***	***p***	**LLCI**	**ULCI**	**Effect**	***SE***	***t***	***p***	**LLCI**	**ULCI**
Total effect	−0.58	0.07	−7.83	<0.001	−0.73	−0.44	−0.24	0.05	−2.82	0.005	−0.40	−0.07
Direct effect	−0.35	0.07	−5.27	<0.001	−0.49	−0.22	−0.06	0.05	−0.68	0.497	−0.22	0.11
Total indirect effect	−0.23	0.04			−0.32	−0.15	−0.18	0.04			−0.27	−0.10
Indirect (X→*M*_1_→Y)^a^	−0.06	0.03			−0.12	−0.02	−0.03	0.02			−0.08	0.01
Indirect (X→*M*_2_→Y)^a^	−0.13	0.03			−0.20	−0.07	−0.12	0.03			−0.19	−0.06
Indirect (X→*M*_1_→*M*_2_→Y)^a^	−0.03	0.01			−0.06	−0.01	−0.03	0.01			−0.06	−0.01

a *based on 5,000 bootstrap samples. LLCI, Lower Limit 95% Confidence Interval; ULCI, Upper Limit 95% Confidence Interval*.

The total effect of disagreeableness on dominance was also significant (*b* = −0.24, SE = 0.05, *t* = −2.82, *p* = 0.005, 95% CI [−0.40, −0.07]) but became non-significant when depression stigma and empathic concern were entered into the model (*b* = −0.06, SE = 0.05, *t* = −0.68, *p* = 0.497, 95% CI [−0.22, 0.11]), suggesting full mediation. Stigma and empathic concern individually (H2-H3) and serially (H4) mediated the association between disagreeableness and dominance (see Figure 4).

The goal of Study 2 was to replicate the findings from Study 1 in a community sample. Similar to Study 1, disagreeableness was associated with less compassionate and more rejecting behaviours. Disagreeable individuals reported more critical and distant behaviours toward depressed targets. While disagreeableness was negatively correlated with love across both studies, there was also a negative association between disagreeableness and dominance in Study 2. Depression stigma and empathic concern individually and serially mediated the effects of disagreeableness on loving and dominant support. The results showed that disagreeable individuals were more likely to endorse stigmatic attitudes toward depression, and that stigma in turn, was associated with less empathic concern. These mediators accounted for less loving and less dominant behaviours reported toward depressed targets, in support of our hypotheses.

## Discussion

Interpersonal theories of depression emphasise the ways in which depressed individuals contribute to the rejection they experience in close relationships (11, 12). These models describe deficits in social skills, and problems with communication that act as putative causal factors for the onset, maintenance, and recurrence of depression (12). The negative interactional cycles studied in dyads with a depressed partner acknowledge reciprocal influences between partners [e.g., (18, 52, 73)]. However, individual differences among support providers have not been studied independently in the literature on depression. Disagreeableness, depression stigma, and empathic concern among significant others were examined here as potential contributors to compassionate and rejecting behaviours toward depressed individuals. Compassionate vs. rejecting forms of support were captured with the loving and dominant dimensions of the interpersonal circumplex.

There were distinct interpersonal patterns reported by disagreeable providers in their relationship with depressed targets. The hypothesis that disagreeableness would entail less loving support was supported across both studies. Disagreeableness was negatively correlated with dominance in Study 2. Disagreeableness was primarily related to more critical and distancing forms of support toward depressed individuals as reported by providers. These findings generally suggest that disagreeable providers were more rejecting toward depressed individuals, consistent with interpersonal models of depression (12). The current work accentuates how maladaptive interpersonal dynamics among depressed individuals are more likely to occur with significant others who are disagreeable. This research also expands on the existing literature on trait agreeableness in a specific context of support provisions toward depressed individuals.

The mediational tests examined depression stigma and empathic concern as proximal predictors of compassion and rejection toward depressed individuals. Depression stigma was associated with less dominance in Study 2 and less love across both studies. Providers who endorsed stigmatic attitudes about depression reported more critical and distancing behaviours toward their depressed significant other. Empathic concern, on the other hand, was consistently associated with more nurturing and engaging support reported toward depressed targets. Providers who felt more empathy for depressed targets reported engaging in more compassionate rather than rejecting behaviours. Overall, the findings highlight the effects of support providers' attitudes (e.g., depression stigma), and emotions (e.g., empathic concern) in the type of support provided to a significant other who is depressed.

Serial mediation models were proposed to determine whether stigma and empathic concern mediated the associations between disagreeableness and social support. The models were tested on love and dominance separately. The results for the love axis were consistent across both samples; stigma and empathic concern partially mediated the relation between disagreeableness and less loving responses toward the depressed target. The indirect effects indicated that the association between disagreeableness and love was serially mediated through greater depression stigma followed by less empathic concern. Although our study does not allow for causal statements, the results are consistent with the proposition that disagreeable providers report more rejecting behaviours toward depressed significant others because of endorsed stigmatic beliefs and reduced empathy.

The tests revealed partial mediation for the love axis, which means that less loving outcomes were not fully accounted for by the mediators. Rejection from disagreeable providers could not be solely attributed to stigmatic attitudes and low empathic concern. This suggests that deficits in compassion associated with this personality dimension may be influenced by other factors. Disagreeableness has a long history in the empirical literature demonstrating traits of interpersonal irritability that may be generalizable across situations (22, 74).

The serial mediational analyses for the dominance axis revealed mixed results. Disagreeableness was not significantly related to dominance in Study 1 but there was a significant negative association in Study 2. The mediation models revealed that disagreeableness predicted less dominant support toward depressed targets through stigmatic beliefs and lower empathic concern in Study 2. Full mediation suggests that disagreeable individuals were more likely to report withdrawing from depressed others because of their stigmatic attitudes and lack of empathy.

It has been previously suggested that trait variables map onto the love axis more strongly than the dominance axis due to situational variability in the provision of social support (44, 75). That is, significant others may be disproportionally more likely to provide nurturing or critical forms of support (i.e., love) toward depressed individuals based on their personality, whereas directive or avoidant behaviours (i.e., dominance) may be more influenced by the context (i.e., attitudes and emotions). It is also worth highlighting that the inconsistent finding regarding disagreeableness and low dominance between our samples may reflect a general measurement issue. The brief measure of disagreeableness used in Study 1 may not have reliably captured the construct of disagreeableness as much as the measure used in Study 2.

In addition to the serially mediated effects, stigma and empathic concern individually mediated the association between disagreeableness and support. Empathic concern proved to be the most salient mechanism predicting compassionate and rejecting outcomes. Consistent with previous research linking agreeableness to empathic concern and prosocial outcomes (22), our results show that the reverse is also true. That is, the absence of empathic concern partly explained the relationship between disagreeableness and more rejecting outcomes toward depressed individuals. Compassionate action involves noticing the suffering of the other in need, empathising with their distress, and attempting to ease their suffering through supportive actions. Without empathic concern, helpful supportive actions are compromised.

Critical forms of support, as measured in the current work, overlaps with the literature on expressed emotion in families of those suffering from depression (76, 77). Studies have revealed higher levels of critical expressed emotion in family members of depressed individuals [e.g., (78)]. Critical forms of expressed emotion within these families has been shown to predict relapse in individuals with a history of the disorder (79) and can have important ramifications for the course of their symptoms. Low levels of social support are generally associated with poorer mental health and increased risk for mortality (80, 81). In the case of depression, criticism from support providers may be particularly pernicious. The current studies have identified how disagreeable traits, stigmatic attitudes, and unemphatic responding among providers may incite more critical forms of expressed emotion toward depressed individuals.

### Clinical Implications and Interventions

Widespread online programs designed to promote literacy about mental health, including depression have shown some promise. For example, a meta-analysis of anti-stigma approaches, including over 38,000 participants from 14 countries, found that public education programs were effective in reducing stigma (38). The programs were found to be more effective than social activism and had significant effects on attitudes, affects, and behavioural intentions toward the mentally ill (38). Community-based interventions with a strong participatory component could also be delivered to promote contact, dispel myths, and provide education about depression. A key aspect of stigma stems from beliefs around the controllability of symptoms (82). Stigmatic attitudes include the belief that depressed individuals are unpredictable, weak, and to blame for their symptoms (38). The alleviation of stigma could lead to a substantial improvement in exercising empathy toward those suffering from depression.

Among depressed participants themselves, a randomised controlled trial examined the effects of a web-based depression literacy intervention compared to a cognitive-behavioural intervention in reducing stigmatic attitudes toward depression (39). Both interventions significantly reduced personal stigma (39). The literacy website condition provided information on the nature of depression and its debilitating consequences, along with descriptions of the most promising forms of treatment. The authors concluded that psychoeducational programmes may be helpful in alleviating stigmatic attitudes although it is not yet clear if such programmes could help reduce stigma held in the general population. Such campaign could be disseminated widely and may be particularly relevant for significant others of those with depression.

It can be argued that stigmatisation is a social problem that does not lie within individuals *per se* (83). Society places a stigma label on an individual suffering from a mental illness while oblivious to other aspects of the individual. These social norms lie within a culture that can be shaped. For example, communal values that are cherished by all can be emphasised and promoted at all levels of a society, including compassion, civility, altruism, forgiveness, and harmony [see (84)]. Public campaigns could be successful in promoting these values to effectively increase tolerance, acceptance and understanding toward those suffering from depression. The following paragraphs outline specific intervention targets that could also improve compassionate responding.

Empathy training could have tremendous social outcomes (85). Studies have shown that inducing empathy can produce increased rates of helping (86). Empathic concern can also be promoted through perspective taking exercises. Habashi et al. (22) found that a perspective taking intervention was particularly effective for disagreeable individuals who do not habitually use these social skills. Overall, promoting a empathic understanding around the nature of depressed individuals' suffering could enhance compassionate responding.

In addition to reducing stigma and promoting empathy, interventions can be geared at increasing compassionate action among support providers directly. Considerable amounts of research have shown that performing acts of kindness improves the well-being of the actor (7). There is sizable evidence showing that compassionate behaviours come with physical and psychological benefits for the provider (8). A recent study has shown that disagreeable individuals may have even more to gain from practising compassion (87). Disagreeable individuals doing acts of kindness reported greater reductions in depression, and greater increases in life satisfaction 2 months post-test compared to those in a loving-kindness and placebo control conditions (87). It was suggested that this type of exercise may act as skills training for disagreeable individuals, which could benefit their mood by increasing harmony and compassion in their relationships.

The study of support providers is important because compassionate and rejecting forms of support provided toward depressed individuals have consequences for their long-term outcomes (9). Social support is a vital and necessary part of depression recovery. Higher perceived support prospectively predicts remission from depression (16). Conversely, deficits in perceived social support predict worse outcomes, such as increases in symptoms and new onsets (17, 88, 89).

### Limitations and Future Directions

Data were obtained from support providers but not from the depressed targets themselves. This provides only half the picture among dyads, and precludes a full understanding of the dynamic interplay between support providers and depressed recipients. For example, the targets may have pulled for more rejecting and less compassionate responses if they held stigmatised attitudes toward themselves and viewed their depression as a sign of weakness (90). They may have refrained from asking for support directly, as do individuals who feel stigmatised, using maladaptive and indirect strategies to seek support (90). These strategies may backfire and result in unsupportive responses, feeding into a cycle of rejection for those with personal stigma (40). This is consistent with self-verification theory (21) where those with low self-esteem will seek corroboration of their negative self-views from their interpersonal environment.

Both samples had a preponderance of female participants (72% in Study 1 and 62% in Study 2). Gender differences on the love and dominant axes of the circumplex have been reported in couple interactions involving a depressed spouse [see (52)]. More specifically, depression in husbands was related to reductions in affiliation (i.e., love). Depression in wives tended to alter levels of dominance in the interaction dynamics (52). The existing literature has also demonstrated a potential effect for gender in the reassurance-seeking rejection link with depressed individuals (19). The current studies found that women reported being more loving toward depressed targets. Nevertheless, the findings from the serial mediation models were robust when controlling for gender suggesting that the results hold for men and women. Future research should examine the effects of disagreeableness, stigma, and empathic concern on supportive behaviours toward depressed individuals using gender-balanced samples. It would also be instructive to examine targets themselves to determine if depressed men elicit different types of social support compared to depressed women.

In addition to collecting information from both partners, a limitation of the current work was its cross-sectional design. This precludes conclusions around cause and effect, and the sequence of events in real-time between providers and depressed targets cannot be ascertained. Longitudinal and experimental designs could be used to explore the causal relations between disagreeableness, stigma, empathic concern, and supportive behaviours. Expanding from the retrospective self-report measures used here, experiencing sampling and observational methods would better capture the interpersonal dynamics between depressed individuals and their support providers and those situations characterised by an absence of compassion.

Finally, it is worth highlighting the heterogeneity in the types of relationships reported by participants. In both samples, depressed targets were friends, partners, or family members whom they had known for a relatively long time. It is difficult to estimate the impact of the providers on the depressed targets' mood and symptoms given the varying degrees of closeness shared among them. For example, the effect of rejection among depressed individuals is more pronounced within romantic relationships compared to non-romantic relationships (19). Furthermore, the amount of contact reported with the target varied greatly. While most participants reported interacting daily or a few times a week, some participants had much less frequent contact. It is thus difficult to estimate the actual impact of reported behaviours on the targets. It will be important to examine the effects of disagreeableness, stigma, and empathic concern across various relational contexts including reports from each member of the dyad.

## Conclusion

Depression is one of the most widespread and onerous mental health conditions affecting millions of individuals and their families. The current research was undertaken to provide a broader and more nuanced depiction of the interpersonal milieu of depressed individuals from the perspectives of support providers. Compassionate and rejecting behaviours reported by support providers were influenced by individual differences in disagreeableness, depression stigma, and empathic concern toward depressed individuals. Greater stigma and lower empathic concern further accounted for the rejecting behaviours reported by disagreeable support providers. It may be possible to reduce stigma and increase empathic concern through skills training. It is also possible to teach compassion as an important human strength with immediate and long-term psychological benefits, particularly for those who are disagreeable. The current research offers targets of interventions for support providers to help promote compassion and mitigate rejection toward depressed individuals, which could have consequential effects for both recipients and providers.

## Data Availability Statement

The datasets and Supplementary Materials for this article can be found in online repositories. The names of the repository/repositories and accession number(s) can be found at: https://osf.io/4h9qr/?view_only=1a37d07eb83548f1a19b2810c522cdee.

## Ethics Statement

The studies involving human participants were reviewed and approved by Human Participant Research Ethics Committee, York University. The patients/participants provided their written informed consent to participate in this study.

## Author's Note

Depression is one of the most widespread and onerous mental health conditions affecting millions of individuals and their families. Interpersonal models of depression have documented the ways in which depressed individuals contribute to the negative interaction cycles in their close relationships. A caveat in this literature is the relative neglect of support provider characteristics and the ways in which these may independently contribute to negative outcomes in those relationships. We investigated the support providers of individuals who were depressed and measured personality, depression stigma, and empathic concern. Providers who were disagreeable expressed less compassion and more criticism toward their depressed significant other. Disagreeable providers were also more likely to see depression as a weakness and hold stigmatic attitudes toward the illness. They were less empathic and more critical of a significant other who was depressed. These variables accounted for a significant amount of variance in the type of support provided. We make the case that these provider characteristics should be addressed to promote greater empathic concern and compassionate responding toward individuals who are depressed.

## Author Contributions

All authors listed have made a substantial, direct and intellectual contribution to the work, and approved it for publication.

## Conflict of Interest

The authors declare that the research was conducted in the absence of any commercial or financial relationships that could be construed as a potential conflict of interest.

## Footnotes

^1^One participant reported “not at all” for how frequently they interacted with their depressed significant other. Their open-ended responses revealed that the depressed target was an ex-partner. When omitting the participant from the analyses, the statistical results did not change. Given that interaction frequency was not an explicit exclusion criterion for the study, we decide to keep the participant.

^2^Participants were asked if their significant other endorsed DSM-5 criteria for Major Depression (e.g., “Markedly diminished interest or pleasure in all, or almost all, activities most of the day, nearly every day”) by responding yes, no, or I don't know. The positive responses were specifically summed and averaged to describe depressive severity. The suicidality criterion for Major Depressive Disorder in the DSM-5 was omitted to comply with York University's Research Ethics Board (certificate #e2019-075).

^3^As in Study 1, one participant reported not interacting with their depressed significant other. Closer inspection revealed that they were referring to an ex-partner with whom they currently had limited contact with. Statistical results did not change when the participant was omitted from the analyses. Like Study 1, we decide to keep the participant because interaction frequency was not an explicit exclusion criterion.
