# Supplementary material for: When Depression Breeds Rejection Rather Than Compassion: Disagreeableness, Stigma, and Lack of Empathic Concern Among Support Providers
Source: Front Psychiatry. 2021 Jun 29;12:594229. doi: 10.3389/fpsyt.2021.594229 (PMC8275852; doi:10.3389/fpsyt.2021.594229)
Supplement: Supplementary file 1 [file Data_Sheet_1.docx]

**Study 1. Serial mediation model on loving support with gender and depression as covariates**

Run MATRIX procedure:

***************** PROCESS Procedure for SPSS Version 3.5 *****************

Written by Andrew F. Hayes, Ph.D. www.afhayes.com

Documentation available in Hayes (2018). www.guilford.com/p/hayes3

**************************************************************************

Model : 6

Y : LOV

X : B5_DA

M1 : DS

M2 : EC

Covariates:

G_MF CESD

Sample

Size: 295

**************************************************************************

OUTCOME VARIABLE:

DS

Model Summary

R R-sq MSE F df1 df2 p

.4314 .1861 .5475 22.1802 3.0000 291.0000 .0000

Model

coeff se t p LLCI ULCI

constant 2.1603 .2330 9.2714 .0000 1.7017 2.6189

B5_DA .2663 .0412 6.4612 .0000 .1852 .3474

G_MF -.3210 .0997 -3.2211 .0014 -.5172 -.1249

CESD -.1041 .0734 -1.4180 .1573 -.2487 .0404

Standardized coefficients

coeff

B5_DA .3492

G_MF -.1779

CESD -.0770

Covariance matrix of regression parameter estimates:

constant B5_DA G_MF CESD

constant .0543 -.0063 -.0177 -.0027

B5_DA -.0063 .0017 .0008 -.0003

G_MF -.0177 .0008 .0099 -.0016

CESD -.0027 -.0003 -.0016 .0054

**************************************************************************

OUTCOME VARIABLE:

EC

Model Summary

R R-sq MSE F df1 df2 p

.4044 .1636 1.1735 14.1772 4.0000 290.0000 .0000

Model

coeff se t p LLCI ULCI

constant 6.3894 .3883 16.4563 .0000 5.6252 7.1536

B5_DA -.1988 .0645 -3.0817 .0023 -.3258 -.0718

DS -.3767 .0858 -4.3886 .0000 -.5456 -.2077

G_MF .2461 .1485 1.6572 .0986 -.0462 .5383

CESD -.0678 .1079 -.6280 .5305 -.2801 .1446

Standardized coefficients

coeff

B5_DA -.1809

DS -.2613

G_MF .0946

CESD -.0347

Covariance matrix of regression parameter estimates:

constant B5_DA DS G_MF CESD

constant .1507 -.0092 -.0159 -.0429 -.0075

B5_DA -.0092 .0042 -.0020 .0011 -.0008

DS -.0159 -.0020 .0074 .0024 .0008

G_MF -.0429 .0011 .0024 .0221 -.0032

CESD -.0075 -.0008 .0008 -.0032 .0116

**************************************************************************

OUTCOME VARIABLE:

LOV

Model Summary

R R-sq MSE F df1 df2 p

.6742 .4545 .4728 48.1587 5.0000 289.0000 .0000

Model

coeff se t p LLCI ULCI

constant -.3878 .3427 -1.1316 .2587 -1.0624 .2867

B5_DA -.1391 .0416 -3.3426 .0009 -.2210 -.0572

DS -.4010 .0563 -7.1286 .0000 -.5118 -.2903

EC .2370 .0373 6.3582 .0000 .1636 .3104

G_MF .2534 .0947 2.6756 .0079 .0670 .4398

CESD .0448 .0685 .6544 .5134 -.0900 .1797

Standardized coefficients

coeff

B5_DA -.1613

DS -.3545

EC .3020

G_MF .1241

CESD .0293

Covariance matrix of regression parameter estimates:

constant B5_DA DS EC G_MF CESD

constant .1175 -.0055 -.0098 -.0089 -.0151 -.0036

B5_DA -.0055 .0017 -.0007 .0003 .0004 -.0003

DS -.0098 -.0007 .0032 .0005 .0008 .0003

EC -.0089 .0003 .0005 .0014 -.0003 .0001

G_MF -.0151 .0004 .0008 -.0003 .0090 -.0013

CESD -.0036 -.0003 .0003 .0001 -.0013 .0047

************************** TOTAL EFFECT MODEL ****************************

OUTCOME VARIABLE:

LOV

Model Summary

R R-sq MSE F df1 df2 p

.4747 .2253 .6669 28.2106 3.0000 291.0000 .0000

Model

coeff se t p LLCI ULCI

constant .0672 .2572 .2614 .7940 -.4389 .5734

B5_DA -.3168 .0455 -6.9650 .0000 -.4063 -.2273

G_MF .4691 .1100 4.2648 .0000 .2526 .6856

CESD .0798 .0810 .9851 .3254 -.0797 .2393

Standardized coefficients

coeff

B5_DA -.3673

G_MF .2297

CESD .0522

Covariance matrix of regression parameter estimates:

constant B5_DA G_MF CESD

constant .0661 -.0076 -.0215 -.0033

B5_DA -.0076 .0021 .0010 -.0003

G_MF -.0215 .0010 .0121 -.0020

CESD -.0033 -.0003 -.0020 .0066

************** TOTAL, DIRECT, AND INDIRECT EFFECTS OF X ON Y **************

Total effect of X on Y

Effect se t p LLCI ULCI c_ps c_cs

-.3168 .0455 -6.9650 .0000 -.4063 -.2273 -.3432 -.3673

Direct effect of X on Y

Effect se t p LLCI ULCI c'_ps c'_cs

-.1391 .0416 -3.3426 .0009 -.2210 -.0572 -.1507 -.1613

Indirect effect(s) of X on Y:

Effect BootSE BootLLCI BootULCI

TOTAL -.1777 .0303 -.2392 -.1189

Ind1 -.1068 .0252 -.1593 -.0615

Ind2 -.0471 .0177 -.0846 -.0156

Ind3 -.0238 .0078 -.0412 -.0105

Partially standardized indirect effect(s) of X on Y:

Effect BootSE BootLLCI BootULCI

TOTAL -.1925 .0301 -.2516 -.1335

Ind1 -.1157 .0261 -.1700 -.0681

Ind2 -.0510 .0190 -.0914 -.0169

Ind3 -.0258 .0082 -.0437 -.0116

Completely standardized indirect effect(s) of X on Y:

Effect BootSE BootLLCI BootULCI

TOTAL -.2060 .0325 -.2698 -.1420

Ind1 -.1238 .0278 -.1805 -.0727

Ind2 -.0546 .0205 -.0986 -.0181

Ind3 -.0276 .0087 -.0462 -.0124

Indirect effect key:

Ind1 B5_DA -> DS -> LOV

Ind2 B5_DA -> EC -> LOV

Ind3 B5_DA -> DS -> EC -> LOV

*********************** ANALYSIS NOTES AND ERRORS ************************

Level of confidence for all confidence intervals in output:

95.0000

Number of bootstrap samples for percentile bootstrap confidence intervals:

5000

------ END MATRIX -----

**Study 2. Serial mediation model on loving support with gender and age as covariates**

Run MATRIX procedure:

***************** PROCESS Procedure for SPSS Version 3.5 *****************

Written by Andrew F. Hayes, Ph.D. www.afhayes.com

Documentation available in Hayes (2018). www.guilford.com/p/hayes3

**************************************************************************

Model : 6

Y : LOV

X : BFI_DA

M1 : DS

M2 : EC

Covariates:

G_MF age

Sample

Size: 281

**************************************************************************

OUTCOME VARIABLE:

DS

Model Summary

R R-sq MSE F df1 df2 p

.3382 .1143 .5953 11.9211 3.0000 277.0000 .0000

Model

coeff se t p LLCI ULCI

constant 1.6849 .2930 5.7509 .0000 1.1081 2.2616

BFI_DA .2959 .0732 4.0434 .0001 .1519 .4400

G_MF -.3551 .0955 -3.7167 .0002 -.5431 -.1670

age .0109 .0042 2.6008 .0098 .0026 .0191

Standardized coefficients

coeff

BFI_DA .2373

G_MF -.2110

age .1520

Covariance matrix of regression parameter estimates:

constant BFI_DA G_MF age

constant .0858 -.0153 -.0165 -.0007

BFI_DA -.0153 .0054 .0006 .0001

G_MF -.0165 .0006 .0091 .0000

age -.0007 .0001 .0000 .0000

**************************************************************************

OUTCOME VARIABLE:

EC

Model Summary

R R-sq MSE F df1 df2 p

.4064 .1651 1.2328 13.6481 4.0000 276.0000 .0000

Model

coeff se t p LLCI ULCI

constant 7.2633 .4461 16.2825 .0000 6.3851 8.1414

BFI_DA -.3880 .1084 -3.5797 .0004 -.6014 -.1746

DS -.4532 .0865 -5.2411 .0000 -.6234 -.2830

G_MF -.1600 .1409 -1.1359 .2570 -.4373 .1173

age .0063 .0061 1.0311 .3034 -.0057 .0183

Standardized coefficients

coeff

BFI_DA -.2102

DS -.3063

G_MF -.0643

age .0593

Covariance matrix of regression parameter estimates:

constant BFI_DA DS G_MF age

constant .1990 -.0279 -.0126 -.0386 -.0014

BFI_DA -.0279 .0117 -.0022 .0005 .0002

DS -.0126 -.0022 .0075 .0027 -.0001

G_MF -.0386 .0005 .0027 .0198 .0000

age -.0014 .0002 -.0001 .0000 .0000

**************************************************************************

OUTCOME VARIABLE:

LOV

Model Summary

R R-sq MSE F df1 df2 p

.6439 .4146 .4948 38.9533 5.0000 275.0000 .0000

Model

coeff se t p LLCI ULCI

constant -.8465 .3957 -2.1391 .0333 -1.6255 -.0675

BFI_DA -.3412 .0702 -4.8576 .0000 -.4795 -.2029

DS -.2155 .0574 -3.7523 .0002 -.3286 -.1025

EC .2930 .0381 7.6828 .0000 .2179 .3680

G_MF .2334 .0895 2.6086 .0096 .0573 .4095

age .0033 .0039 .8426 .4002 -.0043 .0109

Standardized coefficients

coeff

BFI_DA -.2448

DS -.1929

EC .3879

G_MF .1241

age .0407

Covariance matrix of regression parameter estimates:

constant BFI_DA DS EC G_MF age

constant .1566 -.0153 -.0098 -.0106 -.0172 -.0005

BFI_DA -.0153 .0049 -.0006 .0006 .0003 .0001

DS -.0098 -.0006 .0033 .0007 .0012 .0000

EC -.0106 .0006 .0007 .0015 .0002 .0000

G_MF -.0172 .0003 .0012 .0002 .0080 .0000

age -.0005 .0001 .0000 .0000 .0000 .0000

************************** TOTAL EFFECT MODEL ****************************

OUTCOME VARIABLE:

LOV

Model Summary

R R-sq MSE F df1 df2 p

.4504 .2029 .6689 23.5021 3.0000 277.0000 .0000

Model

coeff se t p LLCI ULCI

constant .6947 .3106 2.2369 .0261 .0833 1.3060

BFI_DA -.5579 .0776 -7.1920 .0000 -.7107 -.4052

G_MF .3101 .1013 3.0626 .0024 .1108 .5095

age .0013 .0044 .2954 .7679 -.0074 .0100

Standardized coefficients

coeff

BFI_DA -.4003

G_MF .1650

age .0164

Covariance matrix of regression parameter estimates:

constant BFI_DA G_MF age

constant .0964 -.0172 -.0185 -.0008

BFI_DA -.0172 .0060 .0007 .0001

G_MF -.0185 .0007 .0103 .0000

age -.0008 .0001 .0000 .0000

************** TOTAL, DIRECT, AND INDIRECT EFFECTS OF X ON Y **************

Total effect of X on Y

Effect se t p LLCI ULCI c_ps c_cs

-.5579 .0776 -7.1920 .0000 -.7107 -.4052 -.6124 -.4003

Direct effect of X on Y

Effect se t p LLCI ULCI c'_ps c'_cs

-.3412 .0702 -4.8576 .0000 -.4795 -.2029 -.3745 -.2448

Indirect effect(s) of X on Y:

Effect BootSE BootLLCI BootULCI

TOTAL -.2167 .0420 -.3048 -.1378

Ind1 -.0638 .0254 -.1204 -.0209

Ind2 -.1137 .0309 -.1776 -.0562

Ind3 -.0393 .0126 -.0665 -.0172

Partially standardized indirect effect(s) of X on Y:

Effect BootSE BootLLCI BootULCI

TOTAL -.2379 .0445 -.3315 -.1541

Ind1 -.0700 .0277 -.1318 -.0237

Ind2 -.1248 .0336 -.1950 -.0620

Ind3 -.0431 .0136 -.0727 -.0194

Completely standardized indirect effect(s) of X on Y:

Effect BootSE BootLLCI BootULCI

TOTAL -.1555 .0294 -.2168 -.1000

Ind1 -.0458 .0181 -.0857 -.0154

Ind2 -.0816 .0221 -.1271 -.0400

Ind3 -.0282 .0088 -.0474 -.0125

Indirect effect key:

Ind1 BFI_DA -> DS -> LOV

Ind2 BFI_DA -> EC -> LOV

Ind3 BFI_DA -> DS -> EC -> LOV

*********************** ANALYSIS NOTES AND ERRORS ************************

Level of confidence for all confidence intervals in output:

95.0000

Number of bootstrap samples for percentile bootstrap confidence intervals:

5000

------ END MATRIX -----

**Study 2. Serial mediation model on dominant support with gender and age as covariates**

Run MATRIX procedure:

***************** PROCESS Procedure for SPSS Version 3.5 *****************

Written by Andrew F. Hayes, Ph.D. www.afhayes.com

Documentation available in Hayes (2018). www.guilford.com/p/hayes3

**************************************************************************

Model : 6

Y : DOM

X : BFI_DA

M1 : DS

M2 : EC

Covariates:

G_MF age

Sample

Size: 278

**************************************************************************

OUTCOME VARIABLE:

DS

Model Summary

R R-sq MSE F df1 df2 p

.3343 .1117 .5956 11.4877 3.0000 274.0000 .0000

Model

coeff se t p LLCI ULCI

constant 1.7085 .2952 5.7877 .0000 1.1273 2.2896

BFI_DA .2925 .0732 3.9933 .0001 .1483 .4367

G_MF -.3513 .0959 -3.6647 .0003 -.5401 -.1626

age .0102 .0043 2.4060 .0168 .0019 .0186

Standardized coefficients

coeff

BFI_DA .2358

G_MF -.2095

age .1416

Covariance matrix of regression parameter estimates:

constant BFI_DA G_MF age

constant .0871 -.0153 -.0167 -.0008

BFI_DA -.0153 .0054 .0006 .0001

G_MF -.0167 .0006 .0092 .0000

age -.0008 .0001 .0000 .0000

**************************************************************************

OUTCOME VARIABLE:

EC

Model Summary

R R-sq MSE F df1 df2 p

.4139 .1713 1.2288 14.1121 4.0000 273.0000 .0000

Model

coeff se t p LLCI ULCI

constant 7.3028 .4492 16.2583 .0000 6.4185 8.1871

BFI_DA -.3874 .1082 -3.5798 .0004 -.6005 -.1744

DS -.4682 .0868 -5.3958 .0000 -.6390 -.2974

G_MF -.1561 .1410 -1.1069 .2693 -.4338 .1216

age .0060 .0062 .9664 .3347 -.0062 .0181

Standardized coefficients

coeff

BFI_DA -.2104

DS -.3154

G_MF -.0627

age .0556

Covariance matrix of regression parameter estimates:

constant BFI_DA DS G_MF age

constant .2018 -.0279 -.0129 -.0389 -.0014

BFI_DA -.0279 .0117 -.0022 .0005 .0002

DS -.0129 -.0022 .0075 .0026 -.0001

G_MF -.0389 .0005 .0026 .0199 .0000

age -.0014 .0002 -.0001 .0000 .0000

**************************************************************************

OUTCOME VARIABLE:

DOM

Model Summary

R R-sq MSE F df1 df2 p

.4270 .1824 .7419 12.1324 5.0000 272.0000 .0000

Model

coeff se t p LLCI ULCI

constant -.5358 .4896 -1.0943 .2748 -1.4998 .4281

BFI_DA -.1261 .0860 -1.4651 .1440 -.2954 .0433

DS -.0995 .0709 -1.4025 .1619 -.2391 .0402

EC .2752 .0470 5.8512 .0000 .1826 .3677

G_MF -.1132 .1098 -1.0305 .3037 -.3294 .1030

age -.0091 .0048 -1.8986 .0587 -.0186 .0003

Standardized coefficients

coeff

BFI_DA -.0877

DS -.0858

EC .3524

G_MF -.0582

age -.1089

Covariance matrix of regression parameter estimates:

constant BFI_DA DS EC G_MF age

constant .2397 -.0231 -.0153 -.0161 -.0260 -.0008

BFI_DA -.0231 .0074 -.0009 .0009 .0005 .0001

DS -.0153 -.0009 .0050 .0010 .0018 -.0001

EC -.0161 .0009 .0010 .0022 .0003 .0000

G_MF -.0260 .0005 .0018 .0003 .0121 .0000

age -.0008 .0001 -.0001 .0000 .0000 .0000

************************** TOTAL EFFECT MODEL ****************************

OUTCOME VARIABLE:

DOM

Model Summary

R R-sq MSE F df1 df2 p

.2121 .0450 .8602 4.3002 3.0000 274.0000 .0055

Model

coeff se t p LLCI ULCI

constant 1.0836 .3547 3.0544 .0025 .3852 1.7819

BFI_DA -.2994 .0880 -3.4019 .0008 -.4727 -.1261

G_MF -.0759 .1152 -.6590 .5105 -.3027 .1509

age -.0098 .0051 -1.9207 .0558 -.0199 .0002

Standardized coefficients

coeff

BFI_DA -.2083

G_MF -.0391

age -.1172

Covariance matrix of regression parameter estimates:

constant BFI_DA G_MF age

constant .1258 -.0221 -.0241 -.0011

BFI_DA -.0221 .0077 .0009 .0001

G_MF -.0241 .0009 .0133 .0000

age -.0011 .0001 .0000 .0000

************** TOTAL, DIRECT, AND INDIRECT EFFECTS OF X ON Y **************

Total effect of X on Y

Effect se t p LLCI ULCI c_ps c_cs

-.2994 .0880 -3.4019 .0008 -.4727 -.1261 -.3172 -.2083

Direct effect of X on Y

Effect se t p LLCI ULCI c'_ps c'_cs

-.1261 .0860 -1.4651 .1440 -.2954 .0433 -.1335 -.0877

Indirect effect(s) of X on Y:

Effect BootSE BootLLCI BootULCI

TOTAL -.1734 .0454 -.2647 -.0867

Ind1 -.0291 .0241 -.0809 .0148

Ind2 -.1066 .0326 -.1730 -.0469

Ind3 -.0377 .0153 -.0722 -.0128

Partially standardized indirect effect(s) of X on Y:

Effect BootSE BootLLCI BootULCI

TOTAL -.1837 .0431 -.2674 -.0975

Ind1 -.0308 .0250 -.0841 .0159

Ind2 -.1129 .0333 -.1799 -.0504

Ind3 -.0399 .0150 -.0723 -.0146

Completely standardized indirect effect(s) of X on Y:

Effect BootSE BootLLCI BootULCI

TOTAL -.1206 .0283 -.1750 -.0640

Ind1 -.0202 .0163 -.0547 .0106

Ind2 -.0742 .0220 -.1184 -.0327

Ind3 -.0262 .0098 -.0476 -.0096

Indirect effect key:

Ind1 BFI_DA -> DS -> DOM

Ind2 BFI_DA -> EC -> DOM

Ind3 BFI_DA -> DS -> EC -> DOM

*********************** ANALYSIS NOTES AND ERRORS ************************

Level of confidence for all confidence intervals in output:

95.0000

Number of bootstrap samples for percentile bootstrap confidence intervals:

5000

------ END MATRIX -----
